# How do immunometabolites shape bacterial infections?

**DOI:** 10.1371/journal.pbio.3003585

**Published:** 2026-01-05

**Authors:** Griffin Gowdy, Alice Prince

**Affiliations:** Department of Pediatrics, Division of Infectious Diseases, Columbia University Irving Medical Center, New York, New York, United States of America; University of Birmingham, UNITED KINGDOM OF GREAT BRITAIN AND NORTHERN IRELAND

## Abstract

Metabolites generated by host and pathogen have a major impact on the severity and outcomes of infection. The metabolic response to infection shapes the nature and intensity of the immune response, both in bloodstream infections and, especially, in the pathogenesis of pneumonia. Some metabolites are closely linked to pro-inflammatory responses, whereas others act as immunomodulators in mitigating damage to the host, a common consequence of inflammation. Immunometabolites are also major factors in driving bacterial adaptation to the host, enabling pathogens acquired from environmental sources to modify their gene expression to optimize for persistent infection. In this era of diminishing antimicrobial efficacy, an appreciation of the immunometabolic responses to bacterial infection may provide novel targets for therapy.

## Abstract 2

Metabolites generated by both host and pathogen have a major impact on the severity and outcomes of infection. This Essay investigates how the study of immunometabolism has yielded insight into the molecular mechanisms underlying bacterial pulmonary infections.

## Introduction

The study of metabolism, the biochemical mechanisms through which organisms transform substrates to harness energy and build structures, started with the mapping of metabolic networks, linking together metabolites through the enzymatic activities that connect them. Armed with these maps, the field branched into myriad directions, yielding tremendous insight into lines of inquiry ranging from the origin of life to the molecular mechanisms underpinning human diseases. Over the past two decades, immunometabolism has emerged as a powerful framework for understanding immune responses, tumor evolution, and pathogen adaptation [1–3]. The analysis of bacterial metabolic activity (which substrates are fermented or oxidized by a given organism) has been the basis for the classification of pathogens for many decades. However, it is now apparent that the metabolic activities of pathogens, as well as those of the host, shape the nature of the immune response and the eventual outcome of an infection.

There has been a striking increase in bacterial infections that arise as a complication of modern medical practices in hosts with underlying conditions. These are often caused by the *ESKAPE* pathogens (*Enterococcus faecium*, *Staphylococcus aureus*, *Klebsiella pneumoniae*, *Acinetobacter baumannii*, *Pseudomonas aeruginosa*, and *Enterobacter spp.*), antibiotic-resistant opportunists typically acquired in healthcare settings [4–6]. Often, these infections are in patients who would otherwise have succumbed to an underlying disease, be it cancer, chronic lung disease, or an autoimmune disorder, but are instead surviving or even thriving as a result of advances in medical practices. The *ESKAPE* pathogens that infect these patients are readily cleared in healthy individuals, yet present challenges in the clinic. Each of these major pathogens has its own metabolic program and degrees of flexibility, giving them distinct advantages in adapting to different niches. An area of particular interest to the growing field of immunometabolism is the lung, in which the study of immunometabolites during pulmonary infection (often by *ESKAPE* pathogens) has helped to explain disease outcomes and identify potential targets for future therapy [3].

In this Essay, we discuss the role of metabolism and immunometabolites in bacterial infection, with a focus on bacterial pulmonary infection. We describe key immunometabolites that regulate the inflammatory tone of the lung and demonstrate their relevance in the pathogenesis of infection and how they drive host adaptation in selected clinically relevant pathogens.

## Immunometabolites and infection

From single cells to complex mammals, organisms at all levels must defend against parasitism and infection in order to traverse their life cycles. Innate and adaptive functionalities of immune systems provide numerous and often redundant mechanisms of surveillance and communication for the complex host to defend itself and maintain homeostasis. The immune system detects pathogens through mechanisms ranging from highly specific to broad in scope: foreign antigens serve as substrates for receptors of the adaptive immune system; immune and stromal cells secrete and respond to cytokines and chemokines; common microbial products activate pattern recognition receptors; and activated host cells produce damage-associated molecular patterns (DAMPs) that elicit a robust, and ideally protective, response [7,8].

In contrast to the adaptive immune response, which is classically highly specific and able to discriminate among novel and previously experienced stimuli, metabolites can function more generally to evoke defense against potential pathogens. The products of metabolic activity, whether involved in oxidative phosphorylation, glycolysis, glutaminolysis, fatty acid oxidation or other major mechanisms to generate ATP at the cellular level, have major effects on immune function, hence the designation “immunometabolites” [1,7]. As these metabolites are secreted or released into the extracellular space, they can affect cells within a given environmental niche and influence the metabolic activity of local and recruited immune cells, either locally or systemically. Moreover, within specific niches, such as in the phagolysosome, immunometabolites such as itaconate can achieve high concentrations, much more so than those achieved in the blood stream [9,10]. Because the kinetics of receptor interactions and chemical reactions involving immunometabolites are dictated in part by local concentration, high compartmentalized abundance can enable biological activities that would not occur at systemic levels.

In the setting of bacterial pulmonary infection, metabolites released extracellularly into the infected airway by phagocytes have a major role in directing bacterial gene expression [3]. Bacteria possess numerous transport systems to import metabolites such as succinate and itaconate, which are potential carbon sources [11,12]. These metabolites, whether of host or pathogen origin, influence the responses of local and recruited immune cells as well as the bacteria themselves. Accumulation of these immunometabolites can push loci of infection toward pro-inflammatory or anti-inflammatory states. The accumulation of specific immunometabolites may yield an excessive pro-inflammatory response, resulting in the production of reactive oxygen species (ROS) and inadvertent host toxicity [13–18]. Alternatively, they may promote a more tolerant response that includes arginase-expressing immune cells such as the myeloid-derived suppressor cells (MDSCs) that produce IL-10 [19,20], a response more tolerant to foreign antigens and less toxic to the host [21–23].

The systemic effects of accumulated immunometabolites, as released from stressed cells undergoing bioenergetic collapse, are best exemplified in severe bloodstream infection or sepsis (Box 1). The immunometabolic hallmarks of sepsis, an overwhelming and dysregulated immune response to microbial contamination of the bloodstream, are well characterized [8]. Immune cells shift into a highly anabolic metabolism, performing aerobic glycolysis to generate energy, reducing equivalents, and molecular building blocks as rapidly as possible in order to effectuate their antimicrobial functions. This swing from homeostasis towards inflammation and pathogen clearance is carried out by the activation of anti-inflammatory pro-resolving immune cell populations with predominantly catabolic metabolisms. Imbalanced pro- and anti-inflammatory programs can result in excessive deployment of immune effectors that destroy host tissues, immunosuppression that enables pathogen proliferation or a futile overlapping of opposing metabolic modes [8].

**Box 1. The immunometabolism of sepsis**

A major dimension of sepsis pathophysiology comprises a dysregulated immunometabolic response. In patients with sepsis, glycolysis produces pyruvate and protons which, in the absence of adequate renal function, results in acidosis and hyperlactatemia [24,25]. Lactate is produced from the reduction of pyruvate by lactate dehydrogenase and can accumulate due to reduced pyruvate utilization due to mitochondrial dysfunction [26] or pyruvate decarboxylase inhibition [27,28], as well as in the context of insufficient hepatic clearance of lactate [29,30]. This upregulation in glycolysis is driven by mTORC1, which promotes HIF-1α expression [31,32]. HIF-1α drives the expression of several glycolytic enzymes as well as the potent and often highly damaging cytokine IL-1β [8]. While an essential component in productive immune responses in many circumstances, excessive IL-1β in terms of abundance or chronicity of production can drive immunopathology [33]. Patients with sepsis often exhibit hypoxemia due to pulmonary damage, which at the cellular level impairs the electron transport chain [34], leading to the accumulation and release of succinate, which further stabilizes HIF-1α and is itself highly pro-inflammatory [35]. Under homeostatic conditions, prolyl-hydroxylases (PHDs) act on HIF-1α, targeting it for ubiquitination and proteasomal degradation. The reaction catalyzed by PHDs converts molecular oxygen and α-ketoglutarate to carbon dioxide and succinate; as a reaction product, succinate competitively inhibits PHD catalysis thereby stabilizing HIF-1α [36–38]. Another important carboxylate in sepsis is β-hydroxybutyrate. While blood concentrations of β-hydroxybutyrate are predictive of survival in patients with sepsis [39], metabolic modes that favor resolution and repair, such as ketogenesis, are improperly regulated [8].

## Immunometabolism and pulmonary defenses

The lung is a common site of infection by a variety of bacterial pathogens. Alveolar macrophages act as sentinels within the lumen of the airway to identify and respond to foreign inhalants as well as to endogenous materials, as the airway is continuously exposed to inhaled foreign material [40]. Alveolar macrophages take up and degrade lipid-rich surfactant coating the alveolar epithelium, cope with limited glucose [41], and rapidly phagocytose foreign antigens, cells, and particulate matter [42]. Their steady-state phenotype is fueled by lipid catabolism and oxidative phosphorylation, which are intrinsically anti-inflammatory [43]. The ability of alveolar macrophages to clear debris without becoming activated is important in maintaining the relative sterility of the lung while avoiding tissue-damaging inflammation [44]. Alveolar macrophages promote immune responses to potential pathogens that are inhaled inadvertently and activate signals of danger for the host [40]. Such signals include the immunometabolites succinate [45], fumarate [46], and itaconate [47,48], each of which drives distinct metabolic and immune responses in both host and pathogen.

**Fig 1:**
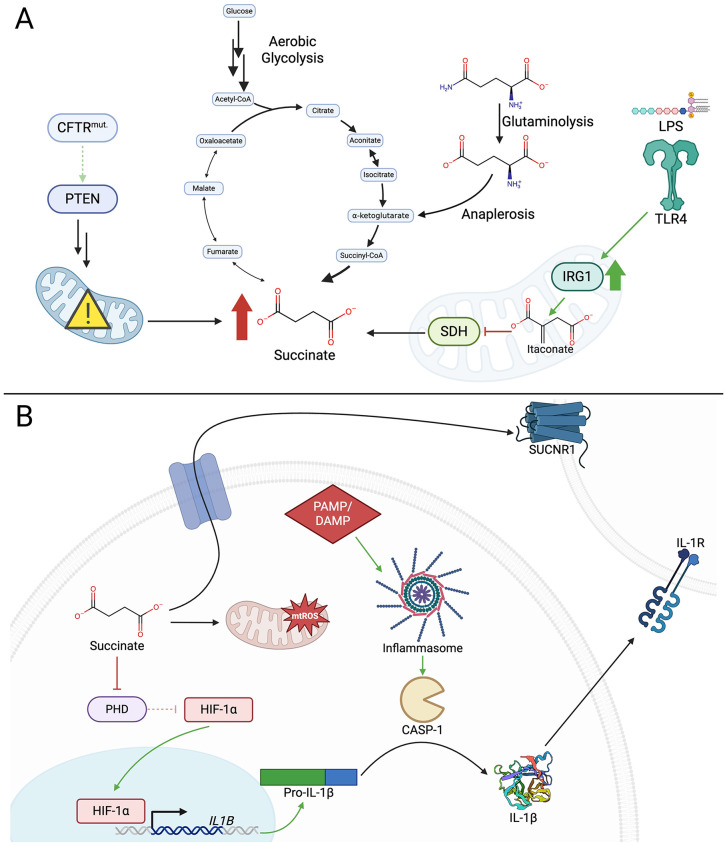
Accumulation and actions of the pro-inflammatory immunometabolite succinate in infection. **(A)** High levels of succinate generation under inflammatory contexts can occur through multiple mechanisms. In cystic fibrosis, the loss of association between the anion channel CFTR and the phosphatase PTEN drives mitochondrial dysfunction and inhibition of succinate dehydrogenase (SDH), resulting in accumulation of succinate [45]. Similarly, pathogen-associated molecular pattern (PAMP) detection by pattern recognition receptors (such as lipopolysaccharide (LPS) detection by TLR4) stimulates expression of *IRG1* in myeloid cells, generating itaconate which inhibits SDH [59,60]. The metabolic pathways engaged by activated immune cells also indirectly result in succinate generation, including aerobic glycolysis which fuels the tricarboxylic acid cycle through pyruvate as well as glutaminolysis and subsequent anaplerosis of glutamate to yield α-ketoglutarate. **(B)** Succinate acts as a pro-inflammatory signal intracellularly and extracellularly. Within cells, succinate can drive the production of reactive oxygen species in mitochondria (mtROS), which includes the superoxide radical and hydrogen peroxide [61–63]. Succinate also inhibits HIF-1α prolyl-hydroxylases (PHDs), resulting in the de-repression of HIF-1α, which subsequently translocates to the nucleus and promotes the transcription of myriad genes including *IL1B* [37,62]. In the presence of a second signal such as a PAMP or damage-associated molecular pattern (DAMP), inflammasome activation can then enable caspase-1-mediated cleavage of pro-IL-1β into its active form, which signals in autocrine, paracrine and endocrine fashions [54]. Succinate can also be exported from the cytosol via members of the monocarboxylate transporter or organic anion transporter families [62,64], and extracellular succinate can signal via the G protein-coupled receptor SUCNR1 [63]. Created with BioRender. https://BioRender.com/6e57t1g.

The environmental bacteria that are aspirated must escape mechanical clearance from the mucociliary escalator to persist in the airway long enough to initiate an infection [49]. Hence, such organisms often possess mechanisms that contribute to pathogenicity and the ability to evade immune clearance. They express a variety of gene products that activate pro-inflammatory signaling, cytokine production, and the release of succinate. Succinate is linked to the hyperinflammatory milieu in the lung that is characteristic of cystic fibrosis (CF). Due to limited interactions of the anion channel CFTR and the phosphatase PTEN [45,50], succinate accumulates both intracellularly and in the airway in patients with CF [45] (Fig 1). Additionally, *P. aeruginosa* lipopolysaccharide (LPS) activates macrophages and stimulates metabolic re-wiring, promoting aerobic glycolysis and the generation of succinate as a consequence of tricarboxylic acid (TCA) cycle interruption [51–53]. Succinate promotes the HIF-1α–IL-1β axis by inhibiting HIF-1α degradation by prolyl hydroxylases [35]. IL-1β itself is highly pro-inflammatory and activates adjacent cells through the ubiquitous IL-1 receptors to also release cytokines [54]. The activation of recruited neutrophils adds to the overall inflammatory response, and their production of proteases and release of toxic oxidants damages both the bacteria and adjacent tissues [55]. In addition to the action of IL-1β indirectly bolstering ROS production, succinate itself directly contributes to mitochondrial production of ROS [53]. Production of reactive oxidants by immune cells is a major antimicrobial mechanism, as exemplified by the susceptibility to infection by *S. aureus* of patients with chronic granulomatous disease, whose phagocytes are unable to generate ROS [56]. However, oxidants also react with host biomolecules and, in excess, can damage membranes, DNA, and proteins of somatic cells [57,58].

As infection progresses into chronicity, microbial variants are selected that are less immunostimulatory, with decreased surface display of LPS, abrogated flagellar biosynthesis, and diminished secretion of toxins [65]. The interdependent alteration in bacterial gene expression within the host and the tolerance of the host for such host-adapted bacteria enables chronic infection [52]. These variants exhibit enhanced production of extracellular polysaccharide (EPS), the matrix component of the biofilm that protects organisms from oxidative stress [66] and impairs opsonization and phagocytosis [67,68]. Organisms that have adapted to form a biofilm either on tissues or on indwelling devices (such as catheters, tracheostomy tubes, heart valves and artificial joints) are a major cause of infections that are recalcitrant to eradication [69,70]. Such host-adapted variants may elicit different immunometabolites, such as less of the pro-inflammatory metabolite succinate but more fumarate and, especially, more itaconate [71].

**Fig 2:**
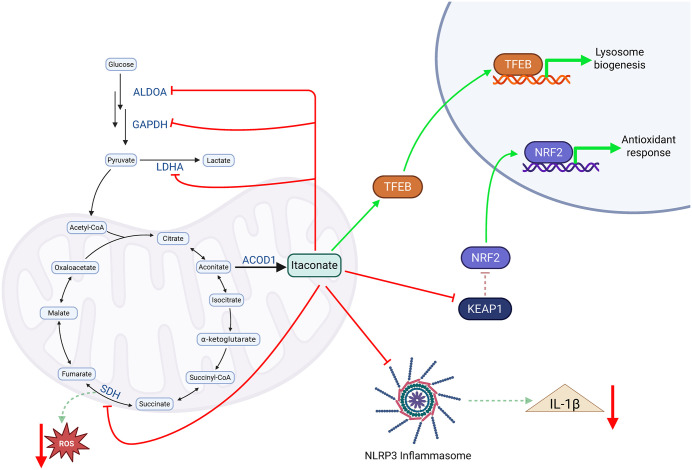
Itaconate polarizes host cells toward an anti-inflammatory phenotype. Itaconate exerts its effects on host cells through multiple mechanisms. It modulates central metabolism by inhibiting glycolytic enzymes aldolase (ALDOA) and glyceraldehyde 3-phosphate dehydrogenase (GAPDH), blocking pyruvate conversion to lactate by lactate dehydrogenase (LDHA), and implementing a break in the tricarboxylic acid cycle through succinate hydrogenase (SDH) inhibition. Itaconate alters the transcriptome by regulating transcription factors such as the lysosomal biogenesis regulator TFEB and NRF2, which promotes the antioxidant response to combat reactive oxygen species (ROS) and electrophilic stress. Another key target of itaconate is the NLRP3 inflammasome, resulting in decreased production of IL-1β [80]. ACOD1, aconitate decarboxylase 1; KEAP1, Kelch-like ECH-associated protein 1. Created with BioRender. https://BioRender.com/6e57t1g.

Itaconate has a major role in defining the nature of the host response to infection, as has been discussed in depth elsewhere [47,48,72–74] (Fig 2). It is derived from the TCA cycle intermediate *cis*-aconitate through the action of aconitate decarboxylase (ACOD1; encoded by *IRG1*) and is produced solely by the host, specifically in immune cells [72]. Itaconate has an α,β-unsaturated carbonyl and is therefore capable of covalently binding to cysteine thiol residues on both bacterial and host proteins [75]. *S*-itaconation of target cysteine residues can result in either gain or loss of function [76]. Through this mechanism and due to its structural similarity and therefore competition with multiple central metabolites at enzyme active sites [59], itaconate exerts many signaling functions. As a weak electrophile it is also directly toxic to many bacteria, causing membrane stress [66]. Itaconate also promotes lysosomal biogenesis through the transcription factor TFEB to support phagocytic activity [77]. Yet, it impairs the action of bactericidal ROS as it stabilizes NRF2 via *S*-itaconation of the repressor KEAP1, enabling NRF2 translocation to the nucleus to drive antioxidant transcriptional programs [78]. In addition to these activities, itaconate redirects metabolic flux by directly interacting with core metabolic enzymes, especially those of the TCA cycle. It competitively inhibits succinate dehydrogenase [59] and targets glycolytic enzymes that would otherwise drive succinate production, aerobic glycolysis, and inflammation in response to infection [60]. In bacteria, itaconate is a major factor in driving bacterial metabolic adaptation to the host, imposing selection for variants that can metabolize substrates via pathways not impacted by itaconate [73,79].

Fumarate, the TCA cycle metabolite produced via the oxidation of succinate, is chemically similar to itaconate and likewise anti-inflammatory [48]. It also activates NRF2 to promote antioxidant pathways and can inhibit pyroptosis through covalent modification of gasdermin D [81]. High cytosolic concentrations of fumarate, however, can be pro-inflammatory, as inhibition of fumarate hydratase (FH) can impair IL-10 production and enhance type I interferon signaling [82].

As in sepsis, activation by pro-inflammatory stimuli causes phagocytes to utilize aerobic glycolysis to fuel their antimicrobial functions. To sustain high rates of glycolysis, NADH is recycled via the reduction of pyruvate to lactate, which is then exported. The effect of lactate on immune cells has recently been reviewed [83]. Mechanistically, lactate can signal to immune cells in multiple ways, including through the G protein-coupled receptors GPR81 and GPR132 [84–86] or through lactylation of proteins, including histones, which modulates gene expression [87,88] and HIF-1α, which drives an M2-like proangiogenic phenotype in macrophages [89,90]. Bacteria can also sense, produce, and degrade lactate [91], as well as manipulate lactate metabolism by host cells [92,93]. Lactate dehydrogenase activity contributes to survival of *P. aeruginosa* phagocytosed by macrophages [94] and to the fitness of *Acinetobacter baumannii* in mice [95]. The role of lactate in directly coordinating virulence traits has also been described, exemplified by the enhancement of *S. aureus* alpha-toxin by lactylation [96] and the upregulation of capsule biosynthesis by lactate in *K. pneumoniae* [97].

## Immunometabolites drive selection for host-adapted pathogens

The opportunists associated with persistent pulmonary infection must have the ability to adapt to local immunometabolites. In contrast to the pathogens associated with generally acute self-limiting infection such as *Streptococcus pneumoniae*, the *ESKAPE* pathogens have substantially greater metabolic flexibility and can adapt to cause less florid yet intractable pulmonary infections that are common in patients with ventilator-associated pneumonias, chronic-obstructive pulmonary disorder, CF, or pulmonary fibrosis. The properties of organisms that make up the lung microenvironment of such chronically infected individuals differ substantially from those of planktonic organisms isolated from the bloodstream during an acute infection. Strains from such acute infections have been typically used in experimental studies of pathogenesis. It has long been assumed that immune pressure is the driving force for the selection of host-adapted chronic isolates. Indeed, the production of EPS, suppression of pili and flagella expression, and diminished immunogenicity of LPS all serve to limit phagocyte activation and contribute to persistent infection [52]. Less well appreciated are the metabolic factors and the ability to withstand both endogenous and exogenous oxidative stresses that are paramount in driving the selection of bacteria that are successful in long-term infection. For example, EPS and biofilm form in response to oxidant stress even in the absence of phagocytes [98]. The metabolic preferences of common pathogens shape the nature of the immune response that is elicited, as illustrated below for a few common healthcare-associated organisms.

### Pseudomonas aeruginosa

*P. aeruginosa* has tremendous metabolic capabilities and readily adapts to the airway/lung milieu, as well as other tissues. Succinate is a preferred carbon source for *P. aeruginosa* and is particularly abundant in the airway of patients with CF, a common site of *P. aeruginosa* chronic infection [99]. The pro-inflammatory signaling cascades associated with excessive succinate generation, including inflammasome activation, IL-1β secretion, the oxidative burst, and the recruitment and activation of leukocytes [100], are damaging to both host and pathogen. There is therefore selective pressure on *P. aeruginosa* to reduce succinate levels in the respiratory tract and to become less immunostimulatory [79]. Phenotypic and genetic analyses of clinical *P. aeruginosa* strains harvested from chronic infections reveal that they have evolved to limit the display of LPS on the bacterial surface and to diminish the expression of cytotoxins and flagella [101], resulting in reduced activation of pro-inflammatory signaling through TLR4 [66]. Much of this adaptive process is a consequence of local immunometabolites such as itaconate [3,45,66].

Itaconate drives the selection for *P. aeruginosa* phenotypes that achieve homeostasis with the host, enabling persistent infection [79]. An immediate metabolic effect of itaconate is mediated by the covalent modification of cysteine residues on the σ^54^ transcriptional regulator RpoN [102], which enhances metabolic flux through pathways relevant to substrates present in the context of infection [103]. In addition, itaconate results in suppression of glucose [66] and succinate [79] consumption through competitive inhibition or *S*-itaconation of catalytic residues of metabolic enzymes and through direct metabolic assimilation of itaconate as a carbon and energy source [66] (**Fig 2**). In the presence of itaconate, *P. aeruginosa* increases its utilization of the Entner–Doudoroff pathway to fuel the TCA cycle with gluconate rather than with glucose through Embden–Meyerhof–Parnas glycolysis [104], consistent with the increased concentrations of gluconate that are observed in airway fluids from patients with CF [105]. Itaconate also induces substantial membrane stress in *P. aeruginosa*, which favors selection for the *mucA22* mutations that are often observed in clinical isolates and that result in upregulated biosynthesis of the EPS alginate [79]. Such host-adapted strains also exhibit decreased synthesis of phenazines, redox-active metabolites of *P. aeruginosa* that can agonize the host aryl hydrocarbon receptor [106], enable bacterial respiration by shuttling electrons [107], and scavenge iron [108].

Longitudinal analyses of *P. aeruginosa* isolates from patients with chronic infection demonstrate how these organisms adapt to and promote the abundance of itaconate in the lung [109–113]. Diverse but clonally related organisms can be isolated from a single patient and reflect distinct metabolic activities, emphasizing the importance of work characterizing bacterial heterogeneity in complex communities and contexts [114]. Some variants retain the immunostimulatory properties associated with acute infection, while other variants display adaptation to itaconate: abundant production of EPSs such as alginate, which act as oxidant sinks by scavenging free radicals [115] while simultaneously providing structure and stability to biofilms, shield bacteria from direct contact with host cells and soluble immunological factors [79]. Such adapted strains drive a shift in the host immune response towards cytoprotective metabolic modes including ketogenesis and glutaminolysis [52]. Thus, itaconate in particular targets both host and pathogen to create a milieu conducive to coexistence in the form of persistent infection.

### Staphylococcus aureus

*S. aureus* is another major pulmonary pathogen frequently associated with persistent infection. This Gram-positive opportunist elicits a neutrophil-dominated immune response, the predominant source of itaconate production in *S. aureus* infection [116]. To avoid the glycolytic blockade imposed by itaconate, *S. aureus* shunts carbon flux through the pentose phosphate pathway, producing EPS to form biofilm matrix [117]. *S. aureus* strains from hospitalized patients with pneumonia demonstrate major changes in gene expression that render these organisms less immunostimulatory and more able to cause protracted infection [118]. These clinical isolates often express mutations that result in downregulation of *agr* expression, a locus responsible for the control of many genes involved in pathogenesis such as toxins and adhesins [119]. *S. aureus* strains that persist within tolerant hosts often suppress the expression of pro-inflammatory gene products while upregulating those involved in immunosuppression or that impart resistance to antibiotics [120].

*S. aureus*, like *P. aeruginosa*, exhibits substantial metabolic flexibility [121]. Analysis of longitudinal isolates from young patients with CF illustrate metabolic adaptation to collagen and especially to proline, a major component of collagen [122]. In a setting of inflammatory lung damage, fibroblasts lay down collagen to maintain the structural integrity of the tissue, which in excess leads to fibrosis and a decline in tissue function [123]. Collagens, which are composed of glycine, proline, and hydroxyproline residues, comprise the major structural proteins of the extracellular matrix. Activated fibroblasts in the process of collagen production generate ample proline [124]. *S. aureus* preferentially consumes glucose and, in settings where multiple substrates are accessible, the choice of substrates is directed by the carbon catabolite repression (CCR) system [125,126]. This is a common bacterial regulatory mechanism that directs the utilization of the most favorable substrate. While functionally analogous, CCR systems are mechanistically diverse across microorganisms; common signaling motifs include small RNAs, cyclic nucleotides, and protein kinases [127]. CCR directs *S. aureus* proline catabolism in the lung due to the low abundance of glucose [41]. The CCR system of *S. aureus* clinical isolates directs bacteria to consume proline rather than glucose, which precludes competition with infiltrating neutrophils, a population of cells that avidly consumes glucose [122,128–133]. Moreover, these clinical isolates of *S. aureus* stimulate the production of collagen by fibroblasts as well as proteolytic activity that releases proline [122]. The net result of this metabolic adaptation to the lung is the selection of organisms that are less immunogenic and better tolerated by the host, the properties of bacteria that cause persistent infection.

Fumarate metabolism is another important facet of the pathophysiology of *S. aureus* pneumonia [46,121]. In response to fumarate, which impacts both glycolysis and the TCA cycle, *S. aureus* increases FH activity to convert fumarate into malate which, unlike fumarate, lacks an electrophilic alkene [46]. FH activity is enhanced in the presence of itaconate, illustrating that not only is fumarate flux an important regulatory node in the host [82], but also in the microbe [46]. Fumarate itself is anti-inflammatory and thus a fumarate-rich environment elicits immune cells that are more permissive of *S. aureus* [48]. The cumulative effects of host and bacterial regulation of fumarate generate a milieu that contributes to the homeostasis of persistent *S. aureus* infection in the lung.

### Klebsiella pneumoniae

Immunometabolites also have a major role in selection of host-adapted *K. pneumonia* lineages. Of particular concern are the ST258 strains, multiply antibiotic-resistant organisms associated with global outbreaks of infection, especially in the setting of intensive care units [134,135]. Accumulating evidence supports the importance of itaconate in the selection of this epidemic sequence type via the generation and reinforcement of an immunotolerant milieu [136]. In contrast to “classical” or hypervirulent *K. pneumoniae* clones (hv*Kp*), antibiotic-resistant host-adapted strains such as the ST258 strains cause less fulminant but persistent infection [137]. Thus, despite the relative indolence of infections caused by such strains, they impose substantial morbidity and mortality [134,135,138–140]. Although ST258 and the hv*Kp K. pneumoniae* strains synthesize identically immunogenic LPS [141], the net metabolic response of the host to ST258 infection is entirely distinct from hv*Kp* strains such as the bloodstream isolate KPPR1 [142]. ST258 infection promotes the usage of fatty acid oxidation and glutaminolysis in host cells for energy generation, which skew the immune response toward the recruitment of MDCSs [136]. A similar MDSC response is associated with foreign body infections caused by some staphylococcal strains [22].

The term MDSC encompasses multiple heterogeneous populations of cells with distinct ontological origin but similar phenotype properties: they are poorly phagocytic, secrete arginase, and express IL-10, promoting an anti-inflammatory response tolerant to infection [19,22,23,137,143]. IL-10 is not exclusively beneficial to the pathogen, as its absence can impair bacterial clearance and result in exacerbated tissue damage [144]. MDSCs impair T cell proliferation by altering lymphocyte tryptophan metabolism in response to hv*Kp* isolates [145]. MDSCs have also been implicated in promoting *S. aureus* biofilm persistence in a fashion regulated by a glycolysis–HIF-1α axis [21]. Ongoing studies of these myeloid cells are likely to yield helpful insights into the pathophysiology of infection by multiple *ESKAPE* pathogens that exploit immunometabolites to drive host tolerance and enable persistent infection.

### Intracellular pathogens

In contrast to *P. aeruginosa* or *K. pneumoniae*, which are predominantly extracellular pathogens, some organisms, such as the *Mycobacteria* or *Salmonella*, are highly adapted to thrive within eukaryotic cells. *Mycobacterium tuberculosis* (*Mtb*) is an intracellular pathogen, subject to the conditions of its host. Itaconate is an integral component of the host response to *Mtb*, suppressing proliferation through the inhibition of central carbon metabolism at multiple points, as with *P. aeruginosa* and *S. aureus* [78]. *Mtb* possesses the enzymatic machinery required to degrade itaconate, and mutants deficient in such activity are impaired in causing infection [146]. Indeed, mice incapable of itaconate biosynthesis are more susceptible to infection by *Mtb* [146], illustrating the importance of specific immunometabolites in host defense.

While some intracellular bacterial pathogens live freely in the cytoplasm, many reside in subcellular compartments derived from phagosomes [147]. Mitochondrially-produced itaconate is delivered to such phagosomal compartments to kill compartmentalized pathogens such as *Legionella pneumophila* [148] and *Salmonella enterica* serovar Typhimurium [9,149]. Although *Salmonella* is not a typical pulmonary pathogen, its metabolic activity that enables persistence within human phagocytes is especially well characterized [150,151]. Moreover, its specific targets for itaconate have been mapped and their impact on purine metabolism provide a paradigm illustrating the complexities of host–pathogen interactions [102]. It is important to appreciate that *Salmonella* also have the ability to thrive as extracellular pathogens in a setting with substantially less itaconate [152]. Direct exposure of intracellular bacteria to itaconate is complemented by itaconate-promoted TFEB activation, which drives lysosomal biogenesis and thus construction of potent bactericidal products [153]. The study of host–pathogen immunometabolic interactions in infection with intracellular bacteria is an experimentally challenging yet exciting frontier of research likely to garner new insights into medically relevant microorganisms.

## Conclusions and future directions

The pathogens discussed in this Essay provide a few examples of how immunometabolites contribute to immune defenses against bacterial infection. Local abundance of specific metabolites direct immune responses and define environments available to colonizing organisms [154]. In oncology, the importance of tumor metabolic activity has long been appreciated and developed as a target for therapy, both to limit tumor growth directly and to enhance the host immunometabolic responses to specific tumor types [2]. Just as therapies for specific tumors have been developed to exploit specific metabolic activities, manipulation of metabolic pathways could be envisioned to prevent or eradicate infection, particularly those caused by the metabolically versatile *ESKAPE* pathogens. However, targeting the metabolic response to infection as an adjunctive therapy has not yet been widely explored, nor have the immunometabolic responses to specific bacterial infections been well characterized, something that will be crucial in defining the goals of such a therapy. Nonetheless, it is apparent that novel approaches to treating bacterial infections are urgently needed [155,156] and, although the concept of manipulating immunometabolic signals to control or prevent infection is at present mostly theoretical, it does hold promise.

Targeting specific metabolites will be challenging. Acute inflammatory responses, such as those mediated by aerobic glycolysis and activation of the inflammasome in many settings generates so much host-damaging IL-1β that inhibition of this cascade enhances bacterial clearance [157]. The concept of titrating pro-inflammatory signaling in the setting of infection is under active investigation [158,159]. There are substantial efforts to develop congeners of itaconate for therapeutic use in autoimmune, cardiac, neurological, and other diseases in which excessive inflammation is pathological [160]. The anti-inflammatory activities of adenosine, generated by the ectonucleotidases CD39 and CD73 [161], have been studied for therapeutic potential [162]. Adenosine is especially important in the lung, which displays many types of purinergic receptors for this nucleoside [163]. However, although the anti-inflammatory properties of adenosine may help to protect the host, many bacteria readily metabolize adenosine as a carbon source [164,165]. In the setting of staphylococcal pneumonia, the availability of exogenous adenosine deactivates macrophage antimicrobial functions and actually promotes infection [166,167]. Thus, the therapeutic potential of many immunometabolites may be highly dependent upon the nature of the pathogen.

The management of sepsis perhaps best illustrates the attraction and challenges of such host-directed therapeutic approaches. The cytokine storm and excessive inflammation characteristic of septic shock, accompanied by mitochondrial failure and acidosis, are often lethal and make anti-inflammatory therapeutic strategies an appealing approach [168,169]. However, there is also a pronounced immuno-paralysis phase in many patients with sepsis in which immunosuppression is predominant [170]. Therapeutic attempts to further down-regulate already limited immune responses by manipulating host metabolic activity and the delivery of specific metabolites to tissues may not be uniformly beneficial.

A major challenge remains in determining when to escalate pro-inflammatory signaling and when to enhance anti-inflammatory signaling. Despite the risk of host damage, perhaps boosting pro-inflammatory signaling along the succinate–HIF-1α axis to eradicate an offending pathogen is, in some cases, appropriate. Likewise, strategies to promote host tolerance of opportunistic pathogens may be powerful, quelling harmful inflammation while allowing an indolent infection to be maintained. Ideally, an optimal immunometabolic milieu could be identified to achieve homeostasis between host and commensal flora, as exists in the uninfected state. There is substantial progress being made to develop markers that reliably predict outcome from infection and sepsis [171]. These could be used to develop treatment algorithms for specific conditions, and the potential for immunomodulation by altering metabolic activity [172]. The use of artificial intelligence to collate the large amount of data from such diverse patient groups to generate diagnostic and treatment targets for immunometabolic interventions is ongoing and may be a central component of treatment in the future [173].

## Abbreviations

CCR: carbon catabolite repression
CF: cystic fibrosis
DAMPs: damage-associated molecular patterns
EPS: extracellular polysaccharide
FH: fumarate hydratase
LPS: lipopolysaccharide
MDSCs: myeloid-derived suppressor cells
PHDs: prolyl-hydroxylases
ROS: reactive oxygen species
TCA: tricarboxylic acid
